# A divergence diagnostic for monitoring the composition of default in rating-migration matrices

**DOI:** 10.1016/j.mex.2026.104135

**Published:** 2026-08-31

**Authors:** Konstantinos Tolikas

**Affiliations:** Sheffield University Management School, University of Sheffield, Conduit Road, Sheffield, S10 1FL, United Kingdom

**Keywords:** Credit risk, Rating migration, Markov chains, Statistical process control, Hotelling $T^{2}$ control chart, Composition monitoring, Default rate, Procyclicality

## Abstract

Reduced-form credit-portfolio models monitor risk through a single aggregate default rate, the exposure-weighted mean of origin-specific default rates across rating grades, sectors, or regions. That aggregate is only the level coordinate of the default-rate configuration: an origin-asymmetric shock that raises one origin’s default rate and lowers another’s by an offsetting amount leaves the aggregate unchanged while the composition of default reorganizes beneath it. This article describes a method to monitor that missing coordinate. From a decomposed migration matrix we form a normalized divergence statistic—a scalar for two origins and a vector for many—that is invariant to the common level and responds to composition rotations; we chart it with a scalar control limit and a Hotelling $T^{2}$ control chart on the general-K vector, bound the forecast bias a stale matrix imparts to probability of default, expected credit loss, and economic capital, and sign the rotation through a structural Merton model. We set out the two distinct central limit theorems the diagnostic rests on—one cross-sectional, governing the control limits, one temporal, governing the structural regime—and show that under a regime-switching Merton specification the reverse-hazard rate is an inverse Mills ratio, so that the direction of the rotation is itself regime-dependent. The method runs on the segment- or rating-decomposed default data institutions already report.

•A normalized divergence statistic isolates the composition of default that the aggregate rate ignores.

•Scalar and Hotelling $T^{2}$ control charts detect, time, and attribute origin-asymmetric default rotations.

•A closed-form reverse-hazard rate signs the rotation and makes its direction regime-dependent.

## Graphical abstract

**Figure:**
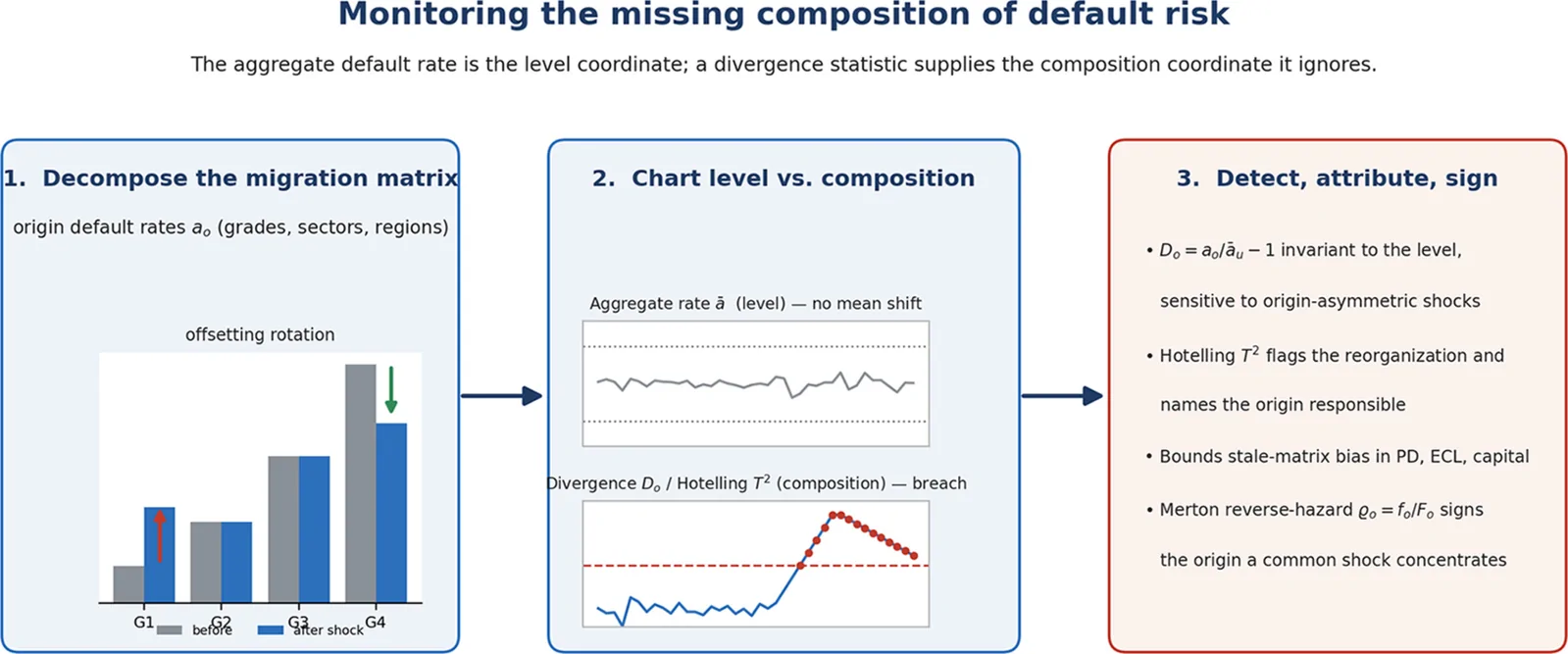

## Specifications table

This table provides general information on your method.

**Table:** 

**Subject area**	Economics and Finance
**More specific subject area**	*Credit risk measurement and monitoring; statistical process control for Markov rating-migration matrices*
**Name of your method**	*The divergence diagnostic: a level–composition decomposition of the default-rate configuration, monitored by scalar and Hotelling*$T^{2}$*control charts*
**Name and reference of original method**	*Markov rating-migration model (Jarrow, Lando & Turnbull, 1997); Hotelling*$T^{2}$*control chart (Hotelling, 1947); Merton structural model (Merton, 1974). The divergence diagnostic and its level–composition decomposition are introduced in this work. The asymptotic foundation draws on the central limit theorem for functionals of jump Markov processes (Huu, Hoang & Ngoc, 2005) and its regime-switching Merton application (Vuong, 2026).*
**Resource availability**	*Public: Federal Reserve commercial-bank charge-off series (FRED); S&P and Moody’s published corporate default studies. Licensed: CRSP and Compustat (WRDS). Replication code, derived data, and a reproduction record accompany the companion article.*

## Background

The risk that credit-portfolio and rating-migration models calibrate, forecast, and watch is an aggregate measure—a portfolio default rate, an average downgrade frequency, or a single migration matrix re-estimated through the cycle—and economic-capital, probability-of-default (PD), and lifetime expected-credit-loss (ECL) figures are built on it. Monitoring the aggregate default rate is sufficient only if the origins of default move together, and the evidence is that they need not: default rates differ across rating grades, industries, and regions and shift with the business cycle [1], and defaults cluster by more than common observable factors explain. A common shock therefore hits some origins harder than others, and the origin default rates can move in opposite directions. When they do, the aggregate default rate can stay flat while the mix of defaults reorganizes, and a manager who watches only that rate sees nothing—so the PD, ECL, and capital figures built on the stale matrix inherit the resulting bias. This method supplies the missing diagnostic: a monitor for the composition of default that the aggregate rate, by construction, cannot register, computed from data institutions already report.

## Method details

**1. The migration model and the default-rate configuration**

Represent a credit portfolio as a Markov chain over K credit states (rating grades, sectors, or regions) plus an absorbing default state, governed by a row-stochastic migration matrix P with sub-stochastic non-default block Q [2]. Partition obligors into K origins; over the monitoring horizon, origin o feeds default at rate $a_{o}$ (its migration-into-default probability). With exposure weights $w_{o}$ ($\sum_{o}w_{o}=1$), the quantity institutions calibrate and monitor is the aggregate default rate $\bar{a}=\sum_{o}w_{o}a_{o}$. Collect the origin rates in the configuration vector $a=(a_{1},\ldots ,a_{K})$. The aggregate is a single linear functional of a; the information it discards is the (K−1)-dimensional set of configurations that share its weighted mean.

**2. The divergence statistic**

Let ${\bar{a}}_{u}=1/K\sum_{o}a_{o}$ be the unweighted mean and define the divergence vector $D_{o}=\frac{a_{o}}{{\bar{a}}_{u}}-1$. For two origins this reduces to the scalar $\Delta =\frac{a_{B}-a_{G}}{a_{B}+a_{G}}$. D is scale-free (invariant to multiplying every $a_{o}$ by a common factor) and responds to any origin-asymmetric change. Its exposure-weighted counterpart, the contribution $C_{o}=\frac{a_{o}}{\bar{a}}-1$, lies in the kernel of the aggregate functional ($\sum_{o}w_{o}C_{o}=0$): under the exposure-weighted inner product it is orthogonal to the level direction. The pair $\left(\bar{a},D\right)$ is a lossless level–composition reparameterization of the configuration a: the aggregate carries the level, the divergence carries the composition.

**3. The aggregation result (why the aggregate misses the rotation)**

Apply an origin-asymmetric shock δ with $\sum_{o}w_{o}\delta_{o}=0$ (for example, raise a_G and lower a_B to hold $\bar{a}$ fixed). The aggregate $\bar{a}$ does not move: an aggregate-rate control chart has no mean shift to detect and fires only at its nominal false-alarm rate, whereas the divergence D moves and a chart on D is built to respond. This is the precise sense in which the aggregate rate is the level coordinate and the divergence the missing composition coordinate.

**4. Charting the scalar (two origins)**

Estimate the in-control mean $\Delta_{0}$ and sampling standard error $\sigma_{\Delta }$ (delta method on the binomial counts) on a calm window, and monitor $\Delta_{t}$ against the control limit $\Delta_{0}\pm \eta \;\sigma_{\Delta }$ (e.g., η=1.96). A breach signals a composition rotation; its sign names the direction. When investment-grade default is a rare-count event, replace the normal chart with a conditional binomial (hypergeometric) tail test on the origin counts, which is the sharper inference.

**5. The general-K Hotelling** $T^{2}$ **chart**

For K>2, monitor the divergence vector with a Hotelling statistic $T_{t}^{2}={\left(D_{t}-D_{0}\right)}^{⊤}\Sigma_{D}^{+}\left(D_{t}-D_{0}\right)$ [3], where $D_{0}$ and $\Sigma_{D}$ are the in-control mean and covariance estimated on a calm window of m periods. Because D lives in a rank-(K−1) sum-zero subspace, $\Sigma_{D}$ is singular and is inverted with the Moore–Penrose pseudo-inverse $\Sigma_{D}^{+}$. Signal when $T^{2}$ exceeds the Phase-II finite-sample upper control limit

$$\text{UCL}=\frac{p\;\left(m+1\right)\left(m-1\right)}{m\;\left(m-p\right)}\;F_{p,\;m-p;\;0.95},p=K-1,$$

where F is the 95th percentile of the F distribution. The chart’s components $D_{o}$ identify which origin moved (attribution). Robustness: re-estimate $\Sigma_{D}$ by Ledoit–Wolf shrinkage toward a scaled identity [4], by its diagonal, and by a moving-block bootstrap of the centred in-control vectors; the major breaches survive every estimator, and choosing the calm window from pooled expansion periods avoids absorbing aggregate-blind episodes into the reference.

**6. Asymptotic foundation of the control limits**

Both charts are asymptotic, and it is worth being explicit about which limit theorem each rests on. The scalar limit of step 4 and the $\chi^{2}/F$ limit of step 5 rest on a cross-sectional central limit theorem for the origin default counts: with $n_{o}$ obligors exposed in origin o, the estimated rates ${\hat{a}}_{o}$ are averages of default indicators, so under a Lindeberg condition and weak within-origin dependence $\hat{a}-a\Rightarrow N\left(0,V\right)$ as $\text{mi}n_{o}n_{o}\to \infty$, and the delta method through the differentiable map $D\left(a\right)=a/{\bar{a}}_{u}-1$ gives $\hat{D}-D_{0}\Rightarrow N\left(0,\Sigma_{D}\right)$ with $\Sigma_{D}=J\;V\;J^{⊤}$ and $J={\bar{a}}_{u}^{-1}\left(I-1/K\tilde{a}1^{⊤}\right)$. Because $\Sigma_{D}1=0$ the covariance has rank K−1, and the pseudo-inverse quadratic form is asymptotically $\chi_{K-1}^{2}$; the Phase-II F limit of step 5 is its finite-sample counterpart when $D_{0}$ and $\Sigma_{D}$ are estimated on m calm periods. The two ways this central limit theorem fails are the reason the robustness apparatus above exists. Default clustering beyond common observable factors inflates V relative to the binomial benchmark, so a naive ${\hat{\Sigma }}_{D}$ is too small and the chart over-fires; the moving-block bootstrap re-estimates the limit without assuming independence. And when investment-grade default is a rare-count event the normal approximation is poor in exactly the tail the chart reads, which is why the conditional binomial (hypergeometric) test of step 4 is the sharper inference there rather than a refinement of it.

A second and distinct central limit theorem operates at the structural layer, accumulating in time rather than in the cross-section. If the solvency process of an origin has a drift modulated by an ergodic jump Markov regime R(t), so that $\text{ln}V_{t}=\text{ln}V_{0}+\int_{0}^{t}\varphi^{*}\left(R(s)\right)\;\text{ds}+\sigma W_{t}$, then $T^{-1/2}\left(\text{ln}V_{T}-\text{ln}V_{0}-\overset{‾}{\varphi }T\right)\Rightarrow N\left(0,\sigma^{2}+\alpha \delta^{2}\right)$, where $\overset{‾}{\varphi }=\alpha \;\Pi \left(\varphi^{*}q^{-1}\right)$ is the ergodic—through-the-cycle—drift constant [5], building on the central limit theorem for functionals of jump Markov processes [6]. The corresponding default approximation $P\left(V_{T}<L\right)\approx \Phi \left(\frac{\text{ln}L-\text{ln}V_{0}-\bar{\varphi }T}{\sqrt{T(\sigma^{2}+\alpha \delta^{2})}}\right)$ supplies an explicit functional form for the origin default probability $F_{o}\left(\theta \right)$ that step 8 takes as primitive. The two limit theorems accumulate in different directions—one in obligors at a date, one in time for an obligor—and neither implies the other: the temporal result does not license the cross-sectional chart, and the chart does not require the structural model. What the temporal result does supply is the regime dependence of the rotation, made precise in step 8, and one caution for the calm-window choice in step 9: a window drawn from expansions estimates a regime-conditional drift rather than the ergodic constant $\bar{\varphi }$, so $D_{0}$ and $\Sigma_{D}$ inherit that conditioning.

The level–composition decomposition also applies to regime-shift tests of the drift itself. A two-sample test of ${\bar{\varphi }}_{\text{stressed}}={\bar{\varphi }}_{\text{normal}}$ built on the temporal limit [5] compares a single drift constant across two windows and is therefore a level monitor: applied origin by origin it produces a vector of drift changes of which it reports only the exposure-weighted mean, so an origin-asymmetric regime shift that leaves that mean unchanged is invisible to it for exactly the reason the aggregate default rate is blind in step 3. The divergence is the composition coordinate of that same vector, and the diagnostic developed here is the extension of such tests to it.

**7. Bounding the stale-matrix forecast bias**

A monitor that re-estimates the matrix only when the aggregate rate moves carries a stale block $\hat{Q}$ in place of the true Q. Through the fundamental matrix $N={\left(I-Q\right)}^{-1}$, the cumulative quantities a manager computes—PD term structures, lifetime ECL, and economic-capital inputs—inherit a bias that is bounded over a finite horizon h by a telescoping of $Q^{\prime h}-{\hat{Q}}^{h}$ (sub-multiplicativity of the sub-stochastic blocks). The divergence chart triggers re-estimation before the aggregate confirms the move, capping this bias.

**8. Signing the rotation (structural Merton condition)**

Embed each origin in a Merton model: obligors of origin o default when the solvency variable crosses a common barrier θ, so that $a_{o}\left(\theta \right)=\lambda_{o}F_{o}\left(\theta \right)$, and a common shock raising θ concentrates default toward the origin with the larger reverse-hazard rate $\varrho_{o}=f_{o}/F_{o}$ [7]. The divergence moves only when the reverse-hazard rates differ over the shock interval, so ϱ signs the rotation. Under the regime-switching normal approximation of step 6 this rate is available in closed form: writing $s_{o}=\sqrt{T(\sigma_{o}^{2}+\alpha_{o}\delta_{o}^{2})}$ for the solvency dispersion and $d_{o}=\left(\mu_{o}-\theta \right)/s_{o}$ for the distance to default, $\varrho_{o}=s_{o}^{-1}\lambda \left(-d_{o}\right)$ with λ(z)=ϕ(z)/Φ(z) the inverse Mills ratio, which is strictly decreasing. Two consequences follow, and they sharpen the usual intuition. First, at equal dispersion the origin further from the barrier carries the larger ϱ: a high-grade origin, precisely because $F_{o}\left(\theta \right)$ is small, responds proportionally more to a common shock. Proximity to the barrier raises ϱ only through the dispersion channel, so an ordering $\varrho_{B}>\varrho_{G}$ requires the low-grade origin to be materially tighter (smaller $s_{B}$), not merely closer to default—and investment-grade participation in a systemic year is therefore the generic prediction of the structural model rather than an anomaly. Second, the ordering is regime-dependent. Under a common volatility scaling $s_{o}\mapsto c\;s_{o}$ one has $\varrho_{o}≍d_{o}/s_{o}$ as c→0 and $\varrho_{o}≍\lambda \left(0\right)/\left(c\;s_{o}\right)$ as c→∞, so a calm regime orders origins by $d_{o}/s_{o}$ and a stressed regime by $1/s_{o}$ alone; when the two criteria disagree there is a critical regime volatility at which the rotation reverses sign. The direction in which the composition rotates is thus a property of the prevailing regime rather than a fixed attribute of the grades, which is what makes it something to be read from the chart rather than assumed.

**9. Implementation (step by step)**

• Input: a decomposed default-rate panel a_{o,t} (segment, grade, or sector × period) — e.g., net charge-off rates by loan segment, annual default rates by rating grade, or debt-weighted Merton expected default frequencies by sector.
• Form the divergence vector $D_{t}=a_{t}/{\overset{‾}{a}}_{u,t}-1$ (or the scalar $\Delta_{t}$ for two origins).
• Choose a calm in-control window (a low-volatility, aggregate-quiet stretch or pooled expansions); estimate $D_{0}$ and $\Sigma_{D}$.
• Compute $T^{2}$_t and the Phase-II UCL; flag periods that breach, and read the leading $D_{o}$ for attribution.
• On a breach, re-estimate the migration matrix and, if desired, rebalance exposure within a fixed-cost no-trade band.

The method requires only the segment- or rating-decomposed default data a bank already maintains; no obligor-level identifiers are needed for the segment-level monitor.

## Method validation

The method was validated on a controlled simulation and three independent panels. In a seeded three-state migration simulation calibrated to a mid-size book, an aggregate-rate chart stays at its 5% nominal size across an offsetting-shock sweep while the divergence chart climbs to near-certain detection and tracks the joint likelihood-ratio benchmark; a twelve-case calibration grid confirms the operating characteristics across default levels, asymmetries, exposure mixes, and monitoring-window sizes. Empirically, on U.S. commercial-bank net charge-off rates (Federal Reserve / FRED, 1991–2026) the Hotelling chart—Phase-II UCL 22.9 on a 2004–06 calm window—flags every major composition reorganization, including ones the aggregate rate ranks as ordinary, with the breaches robust to the covariance estimator. On the S&P and Moody’s annual rating-default histories the scalar divergence reaches its sample minimum in 2008, when investment-grade issuers defaulted while the speculative-grade rate stayed moderate (a conditional count test gives $p\approx 5\times 10^{-15}$). On a Merton distance-to-default panel of >23,000 U.S. firms the contrast reproduces at monthly frequency, with the rotation signed toward financials in 2008 and energy in 2014–16. Complete code and data accompany the companion article [8].

## Limitations

• In books with concentrated debt (e.g., financials) the exposure-weighted contribution $C_{o}$ is dominated by the largest exposure; the scale-free divergence $D_{o}$ is the sharper attribution chart and is the one charted, with $C_{o}$ retained as the loss-tied theory object.
• Credit series are serially persistent, so the normal-theory Hotelling limit is read as a pointwise diagnostic; for sequential monitoring the threshold should be calibrated by the moving-block bootstrap or a horizon false-alarm rule [9].
• The two control limits are asymptotic in the cross-section while the structural regime result is asymptotic in time; a calm window drawn from expansions estimates regime-conditional rather than ergodic quantities, so $D_{0}$ and $\Sigma_{D}$ are conditional on the reference regime, and the bootstrap limit is the safer default when the monitored span crosses a regime change.
• Charge-off and rating panels observe origins at segment or grade level rather than obligor level; the fully exposure-weighted, issuer-level migration-matrix implementation is a direct application of the same diagnostic.
• The Merton distance-to-default is read as a structural risk index (using the risk-free drift) [10], not a calibrated physical default probability; the diagnostic uses only its composition, which is invariant to the common level.
• The fixed-cost rebalancing band is a stylized illustration; a richer loss-minimizing objective replaces it without changing the monitor.
• Empirical magnitudes on FRED charge-offs depend on the data vintage, since the Federal Reserve revises charge-off history.

## Ethics statements

This work did not involve human subjects, animal experiments, or data collected from social media platforms. It uses public-domain aggregate statistics (Federal Reserve / FRED), figures transcribed from published rating-agency default studies (S&P Global Ratings; Moody’s Investors Service), and licensed market and accounting data (CRSP, Compustat) accessed under an institutional subscription. Not applicable.

## Declaration of competing interest

The authors declare that they have no known competing financial interests or personal relationships that could have appeared to influence the work reported in this paper.
